# Canadian Pediatric Neuro-Oncology Standards of Practice

**DOI:** 10.3389/fonc.2020.593192

**Published:** 2020-12-22

**Authors:** Julie Bennett, Craig Erker, Lucie Lafay-Cousin, Vijay Ramaswamy, Juliette Hukin, Magimairajan I. Vanan, Sylvia Cheng, Hallie Coltin, Adriana Fonseca, Donna Johnston, Andrea Lo, Shayna Zelcer, Saima Alvi, Lynette Bowes, Josée Brossard, Janie Charlebois, David Eisenstat, Kathleen Felton, Adam Fleming, Nada Jabado, Valérie Larouche, Geneviève Legault, Chris Mpofu, Sébastien Perreault, Mariana Silva, Roona Sinha, Doug Strother, Derek S. Tsang, Beverly Wilson, Bruce Crooks, Ute Bartels

**Affiliations:** ^1^Division of Neuro-Oncology, The Hospital for Sick Children, Toronto, ON, Canada; ^2^Division of Pediatric Hematology/Oncology, IWK Health Centre, Halifax, NS, Canada; ^3^Department of Oncology, Alberta Children’s Hospital, Calgary, AB, Canada; ^4^Division of Hematology, Oncology and Bone Marrow Transplant, British Columbia Children’s Hospital, Vancouver, BC, Canada; ^5^Department of Oncology, University of Manitoba, Winnipeg, MB, Canada; ^6^Division of Hematology/Oncology, Children’s Hospital of Eastern Ontario, Ottawa, ON, Canada; ^7^Division of Radiation Oncology and Developmental Radiotherapeutics, BC Cancer Centre, Vancouver, BC, Canada; ^8^Division of Pediatric Hematology/Oncology, London Health Sciences Centre, London, ON, Canada; ^9^Pediatric Oncology, Saskatchewan Cancer Agency, Regina, SK, Canada; ^10^Division of Pediatrics, Memorial University, St. John’s, NF, Canada; ^11^Division of Pediatric Hematology/Oncology, Universitaire de Sherbrooke, Sherbrooke, QC, Canada; ^12^Division of Pediatric Hematology/Oncology & Palliative Care, Stollery Children’s Hospital, Edmonton, AB, Canada; ^13^Division of Pediatric Hematology/Oncology, Jim Pattison Children’s Hospital, Saskatoon, SK, Canada; ^14^Division of Pediatric Hematology/Oncology, McMaster Children’s Hospital, Hamilton, ON, Canada; ^15^Division of Hematology/Oncology, Montreal Children’s Hospital, Montreal, QC, Canada; ^16^Division of Hematology/Oncology, CHU de Quebec, Quebec City, QC, Canada; ^17^Division of Pediatric Neurology, CHU Sainte-Justine, Montreal, QC, Canada; ^18^Division of Pediatrics, Queen’s University, Kingston, ON, Canada; ^19^Radiation Medicine Program, Princess Margaret Cancer Centre, Toronto, ON, Canada

**Keywords:** pediatric neuro-oncology, standards, national strategy, low grade glioma, high grade glioma, medulloblastoma, ependymoma

## Abstract

Primary CNS tumors are the leading cause of cancer-related death in pediatrics. It is essential to understand treatment trends to interpret national survival data. In Canada, children with CNS tumors are treated at one of 16 tertiary care centers. We surveyed pediatric neuro-oncologists to create a national standard of practice to be used in the absence of a clinical trial for seven of the most prevalent brain tumors in children. This allowed description of practice across the country, along with a consensus. This had a multitude of benefits, including understanding practice patterns, allowing for a basis to compare in future research and informing Health Canada of the current management of patients. This also allows all children in Canada to receive equivalent care, regardless of location.

## Introduction

Primary central nervous system (CNS) tumors are the 2^nd^ most common cancer and the leading cause of cancer-related death in children (1, 2). Survivors may suffer significant long-term morbidity as a result of the tumor and/or the therapy given (3–6). In general, therapy may consist of surgery, chemotherapy and/or radiation depending on the underlying diagnosis.

Over time, there has been gradual improvement in survival of children with CNS tumors (7). This is related to improved diagnostics, enhanced neurosurgical technique and multimodal therapies. Despite this, a significant portion of patients succumb to their disease and improvements are needed not only with regard to survival but as well with regard to quality of outcome. In order to interpret national outcome data, one must have an understanding of the therapy given. This context is crucial to understanding fluctuations or improvements over time.

When possible, children should be offered enrollment into a clinical trial, however this is not always possible either due to paucity of trial availability, limited spaces for early phase trials or family refusal to participate. Canadian data suggests that only a minority of children with CNS tumors enroll on a clinical trial with the most common reason cited being absence of a clinical trial available (8).

In certain tumors, the molecular drivers have been well described including medulloblastoma (9), pediatric low grade glioma (pLGG) (10), ependymoma (EPN) (11), and atypical teratoid rhabdoid tumor (ATRT) (12). These have been found to have clear implications for outcome; however, clinical trials are just now examining alternative therapies based on molecular subgroup. Historically, patients with one histological diagnosis (i.e. medulloblastoma) have all been treated homogeneously. Targeted inhibitors are also under study in a variety of tumors, though adoption of use outside of clinical trials has been limited in most tumors. In many cases, this molecular information impacts prognosis and trial eligibility, but does not yet alter clinical management in the absence of a trial.

Within Canada, there are 16 institutions that treat children with CNS tumors, all of which are members of C^17^, an organization that unites hematology/oncology/stem cell transplant programs across Canada and represents the interests of children and adolescents with cancer and blood disorders. We sought to create a Canadian national standard of practice for the seven most common pediatric brain tumors, indicating which protocol is considered the standard of care in the absence of a clinical trial. This will help establish important clinical standards allowing all Canadian children to receive equivalent care and provide a basis for essential re-evaluation to advance insight and improve treatment.

## Methods

A survey was sent to all Canadian pediatric oncology institutions to collect data on which protocol would be used in the absence of a clinical trial for common pediatric CNS tumors including pLGG (1^st^ and 2^nd^ line therapy), pediatric high grade glioma (pHGG), childhood and infant MB (localized and disseminated), EPN, ATRT, craniopharyngioma and CNS germ cell tumor (GCT, germinoma and non-germinomatous germ cell tumor (NGGCT)). Tumor molecular information was included where it would potentially impact therapy or management. Prior to finalization, additional details regarding treatment were required and questions for clarification were sent to all institutions. The complete survey is in **Supplementary Figure 1**. In certain tumors, there is equipoise between different regimens, meaning a complete consensus was not necessarily reached in all cases. In this regard, practice patterns were described to understand what the majority of centers were using as standard of care.

The results of the survey formed the consensus statements and subsequently Neuro-oncologists volunteered to prepare a document with a brief introduction and a summary of current practice within Canada. The evolving document was discussed during Canadian National Rounds by experts across the country to ensure agreement. The finalized manuscript was approved by neuro-oncologists across Canada. A summary of the consensus statements can be found in **Table 1**.

**Table 1:** A summary of the consensus statements based on tumor histology and stage.

Low Grade Glioma	*1^st^ Line*	• Vinblastine monotherapy (13)
	*2^nd^ Line*	• Targeted inhibitor if MAPK alteration identified (14, 15), otherwise VCR/Carboplatin (16)
High Grade Glioma		• Chemotherapy/radiation per ACNS0423 (17)
DIPG		• Focal radiation
Infant Medulloblastoma	*AR*	• Chemotherapy per 99703 (18)
	*HR*	• Chemotherapy per 99703 (18) with additional IT chemotherapy
Childhood Medulloblastoma	*AR*	• Radiation/chemotherapy per ACNS0331 with boost to tumor bed
	*HR*	• Radiation/chemotherapy per ACNS0332 Regimen A with boost to tumor bed
Ependymoma	*Localized*	• Focal radiation (19)
Craniopharyngioma		• Intracystic interferon (20)
ATRT		• Chemotherapy per 99703 (18) +/− maintenance chemotherapy, avoid/delay radiation
Germinoma	*Localized*	• Chemotherapy per ACNS1123 Stratum 2 with WVI ± boost to tumor
	*Disseminated*	• Chemotherapy per ACNS1123 Stratum 2 with craniospinal radiation
NGGCT	*Localized*	• SIOP CNS GCT-96 (21) or ACNS0122 (22) with radiation to tumor bed
	*Disseminated*	• SIOP CNS GCT 96 (21) or ACNS0122 (22) with craniospinal radiation

AR, average risk; ATRT, Atypical teratoid rhabdoid tumor; DIPG, diffuse intrinsic pontine glioma; HR, high risk; IT, intrathecal; VCR, vincristine NGGCT, non-germinomatous germ cell tumor; TMZ, temozolomide.

## Results

### Pediatric Low Grade Glioma

*Introduction:* PLGG is the most common CNS tumor in pediatrics. It encompasses WHO grade I and II tumor histologies (23). The 10 year overall survival (OS) is excellent at approximately 85-90%; however, 10 year progression-free survival (PFS) is 42% (5, 24, 25). Patients with neurofibromatosis type I (NF-1) are predisposed to these tumors, primarily optic pathway gliomas (OPG). In the past decade, it has been shown that virtually all pLGG have alterations in the MAPK pathway, with BRAF:KIAA fusion being most common, followed by the BRAF *V600E* mutation (10, 26, 27).

The mainstays of treatment include surgery and chemotherapy, with a radiation-sparing approach whenever possible due to significant long-term toxicity, regardless of age (5). Surgical resection without significant morbidity remains the treatment of choice for surgically accessible tumors. If safe to do so, a gross total resection (GTR) should be performed as this is usually curative. However, GTR of tumors located in the midline is neither feasible nor advisable. Biopsy should be undertaken for pathologic confirmation and molecular analysis when safely feasible. The optimal timing to start chemotherapy is often at the discretion of the treating physician, but should be considered if the patient is symptomatic, or if organ function (i.e. vision) is at risk. There are several chemotherapy regimens considered standard of care worldwide (13, 16). Targeted inhibitors of the MAPK pathway, including BRAF and MEK inhibitors, have been tested in phase 2 trials (14, 15) are currently undergoing study in phase 3 trials (NCT04166409).

Population:

1. Children with pLGG, deemed by the treating physician to require additional therapy following surgery.
2. Definition of pLGG: A variety of histologies including glial or glio-neuronal tumors, generally with a WHO grade I or II designation.

Exclusion Criteria:

1. Patients found to have molecular alterations that would classify them as higher risk, including but not limited to H3 K27M mutation.
2. Higher grade tumors including WHO grade III or IV tumors, or pleomorphic xanthoastrocytoma.

#### First Line Therapy

*Overview:* Vinblastine has been studied in a single-arm phase II trial in the Canadian setting (13). In this trial, vinblastine (6 mg/m^2^) was given once weekly for 70 weeks. Overall, 87% of patients had stable disease, with 25.9% of these patients showing a radiologic response. The 5-year OS was 94.4% (95% CI 88.5% to 100%) and PFS was 53.2% (95% CI 41.3% to 68.5%). The median time to best response was 52 weeks, suggesting prolonged exposure to the treatment regimen is required. These results are comparable to previous trials in pLGG. This treatment is generally well-tolerated with no patients discontinuing treatment due to toxicity. The main toxicity is hematologic with many patients requiring dose reductions, with approximately 1/3 reducing to 5 mg/m^2^ and another 1/3 reducing even further to 4 mg/m^2^. Given this toxicity profile, treatment is usually started with vinblastine at 5 mg/m^2^ dosing at this time. Compared to historical protocols, weekly vinblastine is effective and safe, with a much lower risk of allergic reaction compared to vincristine/carboplatin (28) and fewer long-term side effects compared to the TPCV protocol (16).

***Consensus: If chemotherapy is deemed necessary by the treating physician, there is a complete consensus within C^17^ that single agent vinblastine is the standard therapy for upfront treatment*.**

Treatment Plan:

Vinblastine 5 mg/m^2^ given once weekly for 70 weeks.

For monitoring on therapy, please refer to site-specific guidelines, or to the phase II vinblastine monotherapy protocol for details.

#### Second (and Subsequent) Line Therapy

*Definition of Recurrence/Progression:* Recurrence/progression of pLGG after completion of an initial course of chemotherapy and/or radiologic and/or clinical progression during the first line chemotherapy course.

Repeated relapse/progressive disease in children with pLGG is common in incompletely resected/unresectable tumors. Decision to treat progression or relapse should be made in the context of a multidisciplinary team. Relapse or progression is usually determined by a 25% increase in the tumor (measured by the sum of the product of the two largest perpendicular diameters) and/or clinical deterioration including but not limited to new visual or neurological symptoms. The criterion for 25% progression as an indication for treatment should not be applied to cystic progression which may require surgical intervention.

*Targeted Therapy:* Testing of tumor sample should be done at the time of resection to determine the presence of a molecular alteration. If testing is not available, we recommend sending the tissue sample to a central lab for testing *via* immunohistochemistry/genomic sequencing. Recent phase 2 trials have shown promising results for targeted inhibitors. A phase 2 trial demonstrated 20/25 patients with recurrent or refractory pilocytic astrocytoma harboring either BRAF V600E mutation or KIAA1549:BRAF fusion had either partial response or stable disease with selumetinib (14). Similarly, in patients with recurrent or refractory BRAF V600 mutant pLGG, the 1-year overall response rate was 44% (15).

BRAF fused tumors^*^ - MEK inhibitors such as Trametinib or Selumetinib (14).

BRAF V600E mutation^*^ - BRAF inhibitors such as Dabrafenib or Vemurafenib (15, 29).

***Consensus: Targeted therapy is currently considered as second line treatment for those tumors that harbor alterations in BRAF (14)*.**

In the absence of a trial as the favored choice, access to these drugs is requested *via* the compassionate access program.

*Timing to discontinue targeted inhibitors is unknown. A prolonged treatment plan is recommended. If inhibitors are discontinued due to adverse event/toxicity or another reason, there is a risk of tumor progression, which may be rapid and more aggressive in onset than original presentation. Reinstitution of targeted inhibitor therapy has been shown to achieve a response in this setting.

This may be considered as first line therapy for infant midline gliomas with this mutation, given their historical poor response to chemotherapy (30, 31).

The BRAF inhibitors can be used in combination with MEK inhibitors (trametinib/selumetinib) and this approach can be considered after failure of initial course of BRAF inhibition.

Targeted therapy using MEK inhibitors can also be considered for those patients with NF1 associated optic/suprasellar tumors without tissue confirmation.

*Chemotherapy:* For those recurrent tumors deemed unresectable, or for those tumors that have had an incomplete resection and the clinician’s judgment is to proceed with further therapy given the morbidity of further growth and either no molecular target is identified or patient has either failed or had toxic effects from targeted inhibitors, chemotherapy should be offered. Previous therapy and response should be considered. Second line chemotherapeutic protocols include: weekly vinblastine (if not given as 1^st^ line therapy) (13), carboplatin and vincristine (consensus within C^17^ is that this regimen should be the 2^nd^ line of chemotherapy, if targeted inhibitors are not indicated/possible) (16), carboplatin only, TPCV (the risk of secondary malignancy should be noted with this regimen) (16) and irinotecan and bevacizumab (32).

*External Beam Irradiation:* This modality is generally reserved for older children and for cases where chemotherapy/targeted therapy options have been exhausted.

Though this therapy is curative, the significant long-term morbidities must be considered given the excellent prognosis of these patients. Excess morbidity has been seen in survivors treated with radiation, with a 3-fold increase in death in those who are long-term survivors compared to un-radiated controls (5, 25). Especially, radiation should be avoided in children with NF1 who have even a higher risk of secondary malignancies, neurocognitive challenges as well as vascular sequelae such as Moyamoya syndrome (33).

### Pediatric High-Grade Glioma and Diffuse Intrinsic Pontine Glioma

*Introduction:* Pediatric high-grade gliomas (pHGG) and diffuse intrinsic pontine glioma (DIPG) account for 10%–20% of all pediatric CNS tumors (34). PHGGs and DIPGs are the lead cause of cancer-related death in children under 19 years old (35). Excluding DIPG, pHGG has a 3-year event-free survival (EFS) and overall survival (OS) of ~10 and ~20%, respectively (17, 36, 37). DIPG has a median survival of less than 1 year and is currently not curable (38). The recent identification of an H3.3K27M mutation in the majority of DIPG and several midline pHGG located outside the pons has led to the removal of DIPG from the current 2016 WHO classification of CNS tumors, and the inclusion of diffuse midline glioma (DMG) H3 K27M–mutant as a new distinct entity. However our review of treatment practice distinguished just pHGG and DIPG and did not address DMG separately.

Maximal surgical resection is attempted in pHGG whenever possible. However, DIPG/DMG and many pHGG are not amenable to resection due to critical brain structure involvement by the tumor, while other pHGGs are not fully resectable due to their inherently infiltrative nature. For pHGG, biopsy should be done for pathologic confirmation as well as molecular analysis. Due to significant morbidity of biopsy procedures in the past, treatment for typical DIPG has historically been commenced without histological confirmation, although due to recent identification of distinct mutations and improvement in neurosurgical stereotactic techniques, biopsy is being performed in some centers, and may become routine when therapies specifically targeting these mutations become available. Hence biopsy should be considered in DIPG and importantly in all cases of atypical DIPG – including patients with prolonged clinical symptoms, atypical radiographic findings or outside of the typical age range (i.e. very young and adolescents) to rule out other entities. Focal radiation therapy is the standard therapy used after consideration of biopsy or surgery. Currently, effective chemotherapy regimen for pHGG and DIPG/DMG remain to be determined.

Population:

1. Pediatric patients who are ≥ 3 years of age with pHGG or DMG or ≥ 12 months of age with a DIPG who are treatment naïve.
2. *Definition of pHGG*: Includes a variety of different histologies that are WHO Grade III or IV
3. *Definition of DMG*: H3 K27M-mutant tumors (39).
4. *Definition of a typical DIPG*: Defined clinically (short history duration, and at least two of three classical neurological deficits: cranial nerve deficits, ataxia, and long tract signs) and radiographically to arise from and diffusely involving ≥ 50% of the pons (40).

Recommended Molecular Testing:

Molecular diagnostic pathology should be applied to all childhood glioma including pHGG due to the existence of different subgroups of tumors which might have different prognoses and can respond differently to targeted therapies or immunotherapies. A biopsy should be followed by a molecular analysis, and institutions should attempt to determine the molecular subgroup prior to initiation of chemoradiation if possible.

Specific subgroups important for pHGG include: BRAF-V600E, IDH1 and FGFR mutations for which targeted therapies exist. Pediatric HGG with histone mutations (i.e., DMG H3 K27M or hemispheric pHGG with H3 G34R mutation) are also important for prognostic purposes although targeted therapies are not yet available. Another important subgroup includes those with mismatch repair and hypermutant tumors which will be resistant to temozolomide and sensitive to targeted therapies. Finally, infant HGG often harbor fusions which have specific targeted inhibitors (30, 41).

Exclusion Criteria:

1. Anaplastic ependymoma.
2. Patients with newly diagnosed pHGG that have replication repair deficiency due to germline or somatic alterations should be considered for surgery, +/- focal radiation therapy, and checkpoint inhibitor therapy/trial as applicable.
3. Children with recurrent pHGG.

#### Pediatric High Grade Glioma

*Overview:* Outside of a clinical trial, it has long been recognized that maximal safe surgical resection and focal radiation therapy is standard of care for pHGG (42). CCG-943 randomized patients to surgery, radiation with or without chemotherapy (CCNU, vincristine, and prednisone) where the chemotherapy group appeared to benefit from adding chemotherapy (42). Subsequently, adjuvant chemotherapy became the standard of practice for pHGG and all trials have since included chemotherapy in addition to surgery and radiation.

Many institutions are now using adjuvant temozolomide largely based on adult data (43). However, the addition of temozolomide alone to pHGG therapy did not appear to improve outcomes beyond that of historical, CCG-945, chemotherapy (36). More recently, the addition of bevacizumab to temozolomide did not improve EFS (37), while the addition of CCNU to temozolomide did show an EFS and OS improvement (17). However, the addition of CCNU had significantly more hematologic toxicity than temozolomide alone (17, 36). Given the lack of randomized clinical trials in pHGG, the low number of patients in the initial CCG-943 study showing the benefit of adjuvant chemotherapy, and the lack of clarity around best chemotherapy, a complete consensus for chemotherapy could not be reached.

***Consensus: For pHGG, all C^17^ institutions recommend maximal safe resection followed by focal radiation therapy. In addition, the majority of institutions, 13/16 (81%), are utilizing adjuvant chemotherapy with temozolomide and CCNU as per ACNS0423. The remaining centers treat with focal radiation +/- single agent adjuvant temozolomide*.**

Treatment Plan:

Maximal safe surgical resection, followed by focal fractionated radiation therapy to a dose of 54–59.4 Gy, ideally, within 31 days of the definitive surgical procedure. Chemotherapy is given as per ACNS0423 with 42 days of concurrent temozolomide during radiation therapy at 90mg/m^2^/day followed by a 4 weeks rest then completion of maintenance therapy with six 42 day cycles of CCNU 90mg/m^2^ on Day 1 and temozolomide 160 mg/m^2^/day on days 1–5. A clear goal of maintaining quality of life with careful monitoring for hematologic toxicity with dose modifications, where necessary, is essential. Follow the ACNS0423 protocol for monitoring.

#### Diffuse Intrinsic Pontine Glioma

*Overview:* Outside of a clinical trial, the standard therapy for patients with DIPG remains focal radiation therapy. Numerous prospective clinical trials over the past decades failed to identify a specific chemotherapy with significant prolongation of survival (44–46).

***Consensus: There is complete consensus within C^17^ that focal radiation without chemotherapy is the standard therapy for patients with DIPG*.**

Treatment Plan:

Focal radiation therapy to a dose of 54 Gy.

### Childhood Medulloblastoma (>3–6 years)

*Introduction:* MB is the most common malignant brain tumor of childhood. It is a small round blue cell tumor located in the cerebellum. Overall survival is ~80% for localized disease (47) and ~60% for metastatic disease (48). Analysis of gene expression profiles has subgrouped this tumor into 4 distinct entities including WNT, SHH, group 3 and group 4 (9). These different subgroups have been found to have distinct tumor locations within the cerebellum, unique modifying biomarkers within each subgroup and outcomes vary between subgroups. Hence, current standard treatment is in flux as insight into molecular marker advances and influences clinical risk considerations (49). MB in infants is addressed below as a separate consensus statement, unless under the guise of a clinical trial.

*Overview:* Treatment of MB typically includes surgical resection, radiation and chemotherapy. In terms of surgery, a complete resection as neurologically and surgically safely feasible (less than 1.5 cm^2^) has been associated with a favorable outcome (50). Staging evaluation should include MRI of the spine, ideally prior to resection, post-operative MRI within 72 h of resection to assess for residual disease and lumbar puncture with cytologic assessment at least 14 days following surgery. For radiation therapy, craniospinal irradiation (CSI) therapy is given and varies depending on presence of localized (23.4 Gy) vs metastatic disease (36–39 Gy) with a boost to the tumor bed and site(s) (54–55.8) of disease. The important clinical distinction of average risk (AR) versus high risk (HR) medulloblastoma still persists in the molecular era. AR is defined as a resection with less than 1.5 cm^2^ residual, no metastatic disease on MRI brain and spine and no malignant cells in cerebrospinal fluid (negative CSF) *via* lumbar puncture 10–14 days after surgery. Any result deviating from this definition either a larger amount of residual disease or metastatic findings on MRI or positive CSF is defined as HR. It is recommended that all patients have molecular subgrouping where possible.

WNT MB have an excellent outcome, and efforts should be made to enroll on a trial of de-escalation of therapy for all patients; but de-escalation of radiotherapy should only be done in the setting of a trial. Similarly group 4 MB with whole chromosome 11 loss have an excellent survival as shown in several trials (PNET4, ACNS0331, and SJMB03, unpublished data) validating original finding by Shih et al. (51). Efforts should be undertaken to treat in the setting of a trial. All SHH MB need genetic counseling, irrespective of family history, for evaluation for germline TP53 with consideration of new therapy. WNT MB that do not harbor somatic CTNNB1 mutations require genetic counseling for APC sequencing.

***Consensus: Within Canada, the standard of practice for upfront therapy of AR MB is treatment per ACNS0331 protocol (15/16) and for HR MB is treatment per ACNS0332 Regimen A (13/16). The majority of institutions apply boost to the tumor bed for AR MB (15/16) but five institutions give boost to posterior fossa for HR MB (5/16). For WNT MB all efforts are made to enroll on a trial of de-escalation therapy. Eight of the C^17^ centers will use 36 Gy of CSI in the presence of large cell anaplasia/anaplastic MB irrespective of staging results however this practice is questioned by the other eight centers*.**

*Treatment Plan:* The approach includes upfront surgical resection as deemed safely possible by an experienced neurosurgeon.

**AR MB** are subsequently followed by CSI (23.4 Gy) with a boost to the tumor bed to a total of 54–55.8 Gy with concurrent vincristine, followed by multi-agent chemotherapy with vincristine, CCNU, cisplatin and cyclophosphamide (nine cycles: AABAABAAB per ACNS0331 protocol).

**HR MB** patients are treated with CSI (36–39 Gy) with a boost to tumor bed/posterior fossa to a total of 54–55.8 Gy followed by chemotherapy according to ACNS0332 Regimen A or SJMB-96 (52).

Please refer to the specific protocol for dosing schedule, medication doses and supportive care guidelines (47, 53).

### Medulloblastoma in Young Children

*Introduction*: Medulloblastoma (MB) in early childhood constitutes a significant therapeutic challenge because of the greater vulnerability of the developing brain to cranial irradiation. The definition of “infant brain” or “baby brain tumor” strategies apply to protocols developed for young children with the intent to avoid/delay radiation. The age cut off of this “young children group” is not homogenous throughout the literature. Historically, it referred to children under the age of 36 months but the definition has been extended to older children up to 4 years of age (54, 55) and even up to 6 years of age by other research groups (NCT02875314).

The most common histological subtype of MB in young children is the nodular desmoplastic medulloblastoma (ND MB) or medulloblastoma with extensive nodularity (MBEN), present in 40 to 50% of children under 4 years of age. In young children, the well characterized ND MB/MBEN histological subtype almost exclusively belongs to the SHH molecular subgroup (54, 56, 57). These have been associated with a favorable outcome even for incompletely resected tumors and possibly even in presence of metastatic dissemination (58–60). The prognostic value of methylation subgroups (SHH-β and SHH-γ) still remains unclear and is not currently used routinely to assign treatment risk stratification (54, 56). MB group 3 constitutes the second most common subgroup; the WNT subgroup is excessively rare in young children.

The past 3 decades of clinical trials for young children with MB have explored alternatives to delay or avoid the use of cranial radiation to preserve neurocognitive function. These trials have also brought to light risk factors such as histology and molecular subgrouping based upon which treatment risk stratification has been attempted.

*Overview:* Treatment protocols for infant MB have been based in North America, for the most part, on intensification of therapy with high dose chemotherapy (HDC) and stem cell transplantation (as per CCG 99703; ACNS0334; or Head Start I, II, III, and IV).

*Treatment Plan:* Irrespective of the extent of resection and the metastatic status, histological or molecular classification, the initial overall approach for infant medulloblastoma relies on the following approach in sequence:

1. Maximal safe resection (consider second look surgery). It is recommended that all tumors undergo subgroup analysis where possible.
2. Induction therapy with conventional chemotherapy
3. Consolidation with high dose chemotherapy and stem cell transplantation

In the absence of current consensus, the indication of adjuvant radiation for these patients, is left to the discretion of parents and the treating physicians.

#### Standard Risk Infant MB

The two recommended protocols for upfront therapy for this group of patients are

1. CCG 99703 (18) *
  -. Maximal safe resection
  -. Induction with three cycles of Cisplatin, Vincristine, Cyclophosphamide and Etoposide
  -. Consolidation with three cycles of high dose Carboplatin and Thiotepa
2. Headstart II (61)*
  -. Maximal safe resection
  -. Induction with five cycles of Cisplatin, Vincristine, Cyclophosphamide, Etoposide and High dose Methotrexate
  -. Consolidation with one cycle of high dose Carboplatin, Thiotepa and Etoposide

*Based on limited data, adjuvant irradiation can reasonably be avoided in patients with SHH MB with ND/MBEN histology following these two regimens (59, 62).

Results from ACNS1221 were recently published studying outcomes using the HIT SKK 2000 regimen but omitting intrathecal methotrexate. Although it was a negative trial, patients with MBEN histologies (n=7) had 100% PFS, and this regimen could be considered for a child ≤ 1 year (54). Given the poor inter-observer reliability of MBEN diagnosis, histology should be reviewed and a classic grapelike pattern seen on MRI to consider this approach.

For the other molecular subgroups, the indication of adjuvant irradiation for these patients is left to the discretion of parents and the treating physicians.

***Consensus: CCG 99703 is considered the consensus for treatment in the absence of a clinical trial for radiation-sparing approach within all centers in C^17^*.**

#### High Risk Infant MB

The recommended protocols for upfront therapy for this group of patients are

1. ACNS 0334^+^
  - Maximal safe resection
  - Induction with three cycles of Cisplatin, Vincristine, Cyclophosphamide, Etoposide and High dose Methotrexate
  - Consolidation with three cycles of high dose Carboplatin and Thiotepa
2. Modified CCG 99703^+^
  - Maximal safe resection
  - Induction with three cycles of Cisplatin, Vincristine, Cyclophosphamide and Etoposide with intrathecal chemotherapy
  - Consolidation with three cycles of high dose Carboplatin and Thiotepa
3. Headstart II (61)^+^
  - Maximal safe resection
  - Induction with five cycles of Cisplatin, Vincristine, Cyclophosphamide, Etoposide and High dose of Methotrexate
  - Consolidation with one cycle of high dose Carboplatin, Thiotepa and Etoposide

^+^Indication of adjuvant irradiation for these patients is left to the discretion of the parents and the treating physicians. Consideration can be given to complete tumor response after consolidation chemotherapy, SHH subgrouping and age at treatment completion to guide the decision.

***Consensus: CCG 99703 +/− intrathecal chemotherapy during induction is considered standard of practice at 11/16 centers within C^17^, with the remaining five centers following ACNS 0334*.**

*Additional recommendation:* Given the high prevalence of underlying germline mutation detected in the SHH MB subgroup in young children, patients with ND/MBEN or confirmed SHH MB should be referred to genetic counseling and testing including germline sequencing of *SUFU* and *PTCH*, with all children over age 3 years requiring germline sequencing of *TP53* irrespective of immunohistochemistry (63).

### Pediatric Ependymoma

*Introduction*: EPN, a tumor arising from ependymal glial cells, involves three major anatomic locations (supratentorial brain, posterior fossa and spinal cord) of the CNS and affects both children and adults. Ependymoma accounts for 8%–10% of all childhood CNS tumors and the mean age at diagnosis ranges from 4–6 years with 25%–40% being diagnosed in children less than 3 years of age (64, 65). Survival statistics for ependymoma are generally disappointing with 5-year progression-free and overall survival estimates of 23%–64% and 50%–84%, respectively (19, 66–70). Recurrences are typically local with a median time to recurrence of 13–25 months (66–69, 71); however, 20% of failures have an isolated distant recurrence (72).

Ependymomas form throughout the CNS and are currently sub-divided by three histology-based grades used to predict the natural course of the disease and patient outcome. The World Health Organization (WHO) Grade I tumors include myxopapillary ependymoma (MPE, typically occurs in the spine) and sub-ependymoma (SE-usually intracranial); these tumors are relatively easier to distinguish, occur predominantly in adults and are associated with favorable outcomes with surgery alone (11). Intracranial EPNs occurring in childhood are either WHO Grade II (classic) or WHO Grade III (anaplastic) tumors (11).

Currently, using DNA methylation profiling, EPNs are classified into nine molecular subgroups with distinct clinical and molecular characteristics (73); namely:

1) Supratentorial (ST)- ST-SE (sub-ependymoma), ST-EPN-YAP1 (YAP1 fusion), ST-EPN-RELA (RELA fusion),
2) Posterior Fossa (PF)- PF-EPN-A, PF-EPN-B, PF-SE^*^
3) Spine (SP)- SP-SE (sub-ependymoma), SP-MPE and SP-EPN.

*PF-SE is typically not seen in patients under 40 years of age.

This molecular classification outperforms the current histopathological grading in the risk stratification of patients (11). According to a consensus statement regarding the clinical management of intracranial EPNs, the following were the guidelines released: (a) outside of clinical trials, treatment decisions should not be based on histological grading only (II vs III) but (b) molecular subgrouping should be part of all clinical trials moving forward (11). However, we do acknowledge the limitations of implementing these consensus statements across the C^17^ centers in Canada due to the unavailability of sub-grouping and molecular studies in several centers.

Analysis of data from the recently concluded COG ACNS0121 study shows that ST-EPN with classic histology (WHO Grade II) has excellent outcomes with complete surgical resection without radiation (5 year PFS-61%, OS-100%) (19). The role of adjuvant chemotherapy after first surgery in children younger than 3 years of age to avoid irradiation (Head Start 2/3, POG-9233, ACNS0121) has shown inferior survival when compared to children who receive immediate post-operative RT in the same age group (19, 74). This observation has to be tempered, however in the child under one year of age and the young child with a large ST-EPN tumor, which requires a large CTV (clinical target volume) and high dose radiation in this setting. Conformal radiation therapy (RT) in childhood ST-EPN is associated with high risk of epilepsy (45% in survivors) and disability (10%) (75). Head Start 3 demonstrated 3 year EFS and OS of 86 and 100%, respectively, with an intensive submyeloblative chemotherapy radiation sparing approach for ST-EPN in young children (74).

With existing treatment modalities of radiation and or chemotherapy, there was no prognostic correlation between subgroups in the posterior fossa, prospectively (PF-EPN-A, PF-EPN-B) (19, 76).

The recently concluded COG clinical trial (COG ACNS0831) recommends adjuvant chemotherapy for all patients with sub-total resection (irrespective of histologic grade or location), followed by second look surgery, and involved field radiation. Maintenance chemotherapy is suggested if second look surgery remains subtotal. Patients with ST-EPN tumors with classic histology and a gross total resection-GTR were observed without any treatment. Patients with a localized anaplastic ST-EPN or PF-EPN post GTR were randomized to involved field radiation with or without post radiation maintenance chemotherapy; the results regarding potential superiority of the addition of chemotherapy in terms of survival are yet to be revealed. The benefit of adding chemotherapy (and its related toxicities) in addition to RT in a subgroup specific manner (ST-EPN-RELA, YAP, PF-EPN-A, B) needs to be addressed in prospective clinical trials (SIOP-EPN-II).

Population:

1. Children >1 year of age with histologic/molecular diagnosis of ependymoma

Exclusion Criteria:

1. Children <1 year of age with ependymoma
2. Children with molecular findings inconsistent with ependymoma
3. Children with recurrent ependymoma

***Consensus: In general, outside of clinical trials the standard of practice for intracranial EPNs is maximal safe micro-neurosurgical removal followed by local radiotherapy (54–59.4 Gy) as gross total resection and involved field radiation have consistently demonstrated a survival benefit (16/16). Spinal cord EPNs (SP-SE, SP-MPE and SP-EPN), have an excellent prognosis and are excluded from the recommendation of systematic adjuvant radiation therapy (77)*.**

Treatment Plan:

Spinal EPN (SP-SE, SP-MPE and SP-EPN): Maximal safe surgery alone (77).

ST-EPN: maximal safe surgery followed by focal radiation (19, 78). Albeit histology is generally not recommended for treatment decisions, observation for patients with a GTR and grade two histology could be considered (based on ACNS0121, ACNS0831).

PF-EPN: maximal safe surgery followed by focal radiation (11, 19).

Disseminated EPN (M2/3): Surgery to confirm histologic diagnosis followed by craniospinal irradiation (CSI). This is an infrequent presentation of ependymoma, and diagnosis should be confirmed using molecular markers or methylation classification to exclude alternative diagnosis.

### Atypical Teratoid Rhabdoid Tumors

*Introduction:* Atypical Teratoid Rhabdoid Tumors (ATRT) are highly malignant embryonal brain tumors arising predominantly in very young children (median age of diagnosis 2 years). Historical clinical trials by the Pediatric Oncology Group (POG) 9233/4 and Children’s Cancer Group (CCG) 9921 (79, 80), treated very young children diagnosed with various embryonal brain tumors including ATRTs with multi-agent chemotherapy, resulting in a dismal EFS of 6.4%. Recent multimodal therapeutic approaches including maximal surgical resection and intense chemotherapy/radiation have improved the survival of these patients (81–86).

Population:

1. Pediatric patients of any age with diagnosis of Atypical Teratoid Rhabdoid Tumor (ATRT) who are treatment naïve.
2. *Definition of ATRT*: ATRTs are embryonal brain tumors histologically characterized by cells with rhabdoid features and molecularly characterized by biallelic inactivation of SMARCB1 or SMARCA4. Immunohistochemistry revealing lack of SMARCB1 (INI-1) staining or SMARCB4 (BRG-1) protein expression is the diagnostic criteria used by the World Health Organization (WHO) (39).

Exclusion criteria:

1. Patients with Embryonal Tumors NOS.
2. Patients with recurrent ATRTs.

*Overview:* A multimodal therapeutic approach is necessary for the treatment of ATRTs, although there is still debate regarding the value of each therapeutic component. Gross total resection (GTR) has been associated with better outcomes by many authors (81, 83, 87–91) and maximal safe surgical resection remains the recommended approach in the management of ATRTs. Treatment intensification with high-dose chemotherapy (HDC) and/or radiation therapy (RT) has been a subject of debate over the years. Tekautz et al^13^ published the outcomes of children treated at the St Jude Children’s Research Hospital over a 19-year period noting a significant survival advantage in patients treated with RT. Due to the severe deleterious neurocognitive consequences of RT in the young developing brain, HDC has been adopted by many groups as a RT deferral/avoidance approach (81, 83, 85, 92). Chi et al. published the results of a prospective trial demonstrating the superiority of intensified multimodal therapy (81). Similarly, Quinn et al. (93), reported the benefits of trimodal therapy in a cohort of 190 pediatric patients with ATRTs from the Surveillance, Epidemiology and End Results (SEER) database. The recently published ACNS0333 trial demonstrated improved survival with HDC and RT when compared to historical cohorts (86).

***Consensus: There was consensus regarding the use of high-dose chemotherapy (HDC); all the participating centers are currently using approaches using the COG 99703 (n=12), ACNS0333 (n=3) or ACNS0334+MTX (n=1) backbone. The use of radiation therapy was less clear among the respondents; the majority of centers 93% (n=13) opted for avoiding (n=9) or delaying (n=4) radiation therapy. Additionally, 7/16 centers use a maintenance therapy as described below. This is regardless of dissemination, though one institution would consider adding maintenance therapy for disseminated disease, and two additional centers would consider intensified maintenance for disseminated disease*.**

*Treatment Plan:* Maximal safe surgical resection followed by chemotherapy and potentially followed by maintenance.

Chemotherapy:

1. CCG 99703^*^
  - Maximal safe resection
  - Induction with three cycles of Cisplatin, Vincristine, Cyclophosphamide and Etoposide^*^
  - +/- intrathecal chemotherapy with cytarabine and hydrocortisone
  - Consolidation with three cycles of high dose Carboplatin and Thiotepa
2. ACNS0333^*^
  - Maximal safe resection
  - Induction with two cycles of Vincristine, high-dose Methotrexate, Etoposide, Cyclophosphamide and Cisplatin
  - Consolidation with three cycles of Carboplatin and Thiotepa

*If residual tumor remains, consider 2^nd^ look surgery if feasible prior to embarking on consolidation therapy.

Consolidation: After count recovery, patients will undergo three tandem cycles of HDC using carboplatin and thiotepa and autologous stem cell support.

Maintenance^**^: Patients will receive chemotherapy with oral tamoxifen, 13-cis retinoic acid and intrathecal topotecan for a total of 12 cycles (1 year). An intensified maintenance regimen may be considered in cases of disseminated disease.

**Maintenance therapy has been given in the context of therapy with 99703, and the safety in conjunction with other protocols is unknown.

Radiation: The use of radiation therapy will be assessed on an individual case basis dependent on age, residual tumor, response to chemotherapy and extent of disease.

*Additional recommendation:* Given the significant prevalence of underlying germline mutation detected in SMARC1, patients with ATRT (with or without a renal mass/tumor) should be referred for genetic counseling and testing.

### Craniopharyngioma

*Introduction:* Craniopharyngiomas (CPs) are mixed solid-cystic tumors that account for approximately 5%–10% of brain tumors in children (94, 95) and are one of the most frequently diagnosed hypothalamo-pituitary lesions in this age group (95). They are defined histologically as benign epithelial tumors of the sellar region and postulated to arise from embryonic remnants of Rathke’s pouch during development of the fetal adenohypophysis (96). Although CPs are usually only locally aggressive, treatment is challenging given its anatomical proximity to critical structures surrounding the sellar including the visual pathway, pituitary region, and hypothalamus (97–100). There is a bimodal age distribution with peak ages between 5–14 years and 50–75 years (95). The WHO classifies CPs into two subtypes, adamantinomatous CP and papillary CP, both of which appear to have distinct molecular genetics (23). Children are predominantly diagnosed with the adamantinomatous subtype, which has been associated with CTNNB1 mutations (activation of Wnt signaling pathway), whereas adults typically present with papillary CP, which may harbor BRAF V600E mutations (101).

*Overview:* The general standard curative approach for treatment is safe surgical resection; in case of incomplete resection followed by adjuvant conformal radiotherapy. However, this treatment is often associated with significant morbidity and mortality, and recurrence is still reported despite a complete resection. Children are at risk of neurocognitive, vascular, and endocrinologic sequelae. Hypothalamic sparing is crucial to avoid some of these consequences. There are no systemic chemotherapeutic options that have proven beneficial (102).

On a survey of pediatric Canadian practice, some pediatric institutions have been using intracystic Interferon alpha-2b (IFN) as a first line approach in young children with cystic CP amendable to the safe endoscopic insertion of indwelling catheter(s) and instillation of the drug (103–105). Although the evidence for this is limited, the published reports available demonstrate in their respective cohorts that this can be done safely and can potentially have a treatment benefit in as many as 70% of patients in delaying the need for surgery and radiation (106, 107). The basis of using IFN comes from the premise that CP cyst walls share cells of origin with squamous cell skin carcinomas where this agent is recognized to have anti-proliferative and immunomodulatory properties (108–110). IFN is an inexpensive and non-neurotoxic drug with a tolerable side-effect profile (109). The most common adverse effects (usually grades 1–2) include influenza-like malaise, headaches, fatigue, transient hyponatremia, appetite loss, and weight loss (20). These two approaches are summarized below.

1. Standard treatment approach
  - Hypothalamic sparing safe resection +/− adjuvant RT
  - Consider delaying RT in the following situations: young age, complete resection, no imminent threat to vision and/or pituitary function with progression of disease
2. Alternative treatment approach

Consider intracystic IFN* to delay surgery and/or RT in the following situations:

  - Treating institution has experience with insertion of intracystic catheters and IFN instillation.
  - Young age
  - Cystic component
  - No imminent threat to vision and/or pituitary function with progression of disease

*Consensus: IFN is given to avoid/delay RT in 8/16 centers within C^17^, with another 5/16 using focal RT and the remaining centers basing the decision on patient characteristics.

Treatment Plan:

- Radiation therapy - Conformal photon or proton therapy with 54Gy in 30 fractions
- Intracystic interferon - One cycle = 3,000,000 IU (1mL) M/W/F × 4 weeks (20)

### Central Nervous System Germ Cell Tumors

*Introduction:* Primary intracranial germ cell tumors (GCTs) comprise 3.7% of all primary central nervous system (CNS) tumors in children and adolescents (2) in the Western world and are predominantly located in the pineal and suprasellar regions (111–113). Approximately 60-70% of cases of primary intracranial GCTs consist of germinomas with the remainder being nongerminomatous germ cell tumors (NGGCTs) (112, 114–116). Tumors affecting both the pineal and neurohypophyseal areas are known as bifocal and are more commonly germinomas (113). The incidence of intracranial GCTs is greatest in males, the second decade of life, and in Asia (2, 112, 113, 115). Tumor markers in the serum and lumbar cerebrospinal fluid are important for diagnostic purposes (113). Germinomas can produce small amounts of the β subunit of human chorionic gonadotropin (βHCG) and a level less than 100 IU/L in serum and CSF is accepted in North America (113). The prognosis of germinomas is superior to that of NGGCTs (114, 117). NGGCT’s are a heterogeneous group with several histologies: embryonal carcinoma, endodermal sinus tumor (yolk sac tumor), choriocarcinoma, immature teratoma and teratoma with malignant transformation and mixed tumors, which may include one or more of these histologies as well as germinoma elements.

#### Germinoma

*Overview:* The classification and diagnostic approach to germinomas using tumor markers and the role of surgical biopsy are controversial and outside the scope of this document. Germinomas are both chemosensitive and radiosensitive. With the exception of infants and very young children, all patients with germinomas receive radiotherapy with fields including at least the ventricles (117). For localized germinomas, adjuvant chemotherapy allows reduced radiotherapy doses and is the recommended treatment strategy (117). Therapy decisions should be based on maintaining high cure rates while reducing late effects.

***Consensus: For older children and adolescents with localized germinomas, the ACNS1123 stratum 2 chemotherapy is currently being used at all C^17^ centers followed by radiotherapy 24 Gy whole ventricle irradiation (WVI) for upfront therapy. Three of 16 centers don’t give boost (3/16). Thirteen of 16 centers give a boost to the primary site to a total of 30 Gy (3/16), 36 Gy (7/16) and 40 Gy (1/16) and response dependent (2/16). The study question of whether it is safe to decrease radiotherapy doses to 18 Gy WVI with 12 Gy boost to the primary in the event of CR following chemotherapy has not yet been answered. It is therefore premature to adopt this approach but this decision should be re-assessed following the dissemination of the study’s findings*.**

Treatment Plan:

Recent studies have demonstrated that chemotherapy administered prior to radiotherapy can be used to safely decrease doses of radiotherapy without compromising survival outcomes. Treatment according to ACNS1123 chemotherapy consisting of four cycles of induction chemotherapy with carboplatin/etoposide followed by radiotherapy 24 Gy WVI with or without boost.

For older children and adolescents with **metastatic germinomas**, in the absence of a completed COG study that included patients with metastatic disease, the ACNS1123 stratum 2 chemotherapy with the addition of CSI (24 Gy) is currently being used at 13/16 C^17^ centers.

#### Non Germinomatous Germ Cell Tumors

*Overview:* Platinum based chemotherapy is used in treating NGGCTs and published data has shown that carboplatin has fewer late effects than cisplatin (118, 119) without compromising survival chances. There have been several studies published including SIOP CNS GCT 96 (21), ACNS1123 (120) and ACNS0122 (22).

***Consensus: For NGGCT the SIOP CNS GCT 96 treatment regimen is currently being used at 8/16 C^17^ centers for upfront therapy, utilizing cisplatin with focal tumor bed radiation for those with localized disease and CSI for disseminated disease. ACNS0122 is the standard approach at 5/16 C^17^ centers for localized and disseminated disease*.**

*Treatment Plan:* SIOP CNS GCT 96 is currently being used at 8/16 C^17^ centers, utilizing cisplatin with focal tumor bed radiation for those with localized disease. ACNS0122 is the standard approach at 5/16 C^17^ centers, utilizing carboplatin with CSI for all patients which showed superior overall survival to SIOP CNS GCT 96, though the long-term effects may be more pronounced. Review of resectabality of residual disease after completion of chemotherapy is essential as the difference in overall survival chances drops from 85% in patients without residual disease to 48% in patients with residual (121).

## Discussion

This represents the first standard of practice document for pediatric neuro-oncology within Canada. This was developed considering practice patterns across the country, took into account opinions from experts at each institution and agreed upon prior to finalization. These guidelines apply to the vast majority of pediatric CNS tumors, though not all are represented.

The initial aim of this endeavor are threefold. First was to understand the practice patterns across Canada, understand where this differs and why. While there is certainly clinical equipoise between different therapeutic protocols, the hope is that Canadian children will receive equivalent care across the country, regardless of the institution. The second aim was to help inform future research, including understanding outcomes of patients treated off-study. This must be considered when interpreting survival data. The third aim was to provide a series of recommendations and guidelines as to what is currently considered the standard of care to Health Canada, our national body overseeing healthcare which includes access to non-approved medications and clinical trials.

At a national level, this document has fostered more discussion about our current practices. There were few instances where individual institutions updated or changed their current standard of care based on this review; however, it also led to numerous dialogs regarding what was/was not feasible or accessible in certain centers. This is important to recognize given our diversity and population pattern across the country.

The benefits of this work can also be seen at local institutional levels as well. Certain molecular tests may influence therapeutic decisions. Understanding what molecular testing is offered at other institutions will help individual centers advocate the need to access this at a provincial level. This will help ensure that all Canadian children have access to equivalent work up, tumor analysis and medical care throughout the country.

There are limitations to this project. Firstly, this was developed for treatment considerations in the absence of a clinical trial. Ideally clinical trials would be available and offered to all patients; however, this is not always feasible and trial availability varies across Canada. As a result, these guidelines may be used in a higher proportion of patients in certain areas compared to others. Secondly, these guidelines were developed from current practice patterns and expert opinion. While this is clinically relevant as it represents current best evidence and current practice, a true systematic review was not done to prepare this document. This method is based on evidence presented in the literature and allows for additional nuances to be recognized, but is not as rigorous as a systematic review. Thirdly, these guideline incorporate guidance for medical therapy, including chemotherapy, targeted agents and radiation therapy, but do not address surgical technique. The need for tissue and extent of resection possible should be discussed locally with an experienced pediatric neuro-surgeon in order to safely achieve this. Importantly, it must be noted these standards of practice were developed based on current evidence at the time of survey and may become outdated over time as results of ongoing studies are published. Finally, these results represent current practice across the country. There are scenarios where there are comparable outcomes between various protocols with equivocal outcomes or toxicity profiles. This variability in practice is highlighted in certain consensus statements, emphasizing the diversity across the country.

Moving forward, we hope to use this to understand our patient outcomes. Understanding therapy will allow for better interpretation of historical survival data. This will be reviewed every other year, or in cases of overwhelming practice-altering evidence to ensure it remains clinically relevant. This will continue to be a joint effort from all pediatric neuro-oncologists across Canada.

This work represents the collaboration of pediatric neuro-oncologists across Canada. While the hope was not to homogenize therapy, it allowed for productive discussion and reflection on current practices along with providing more substantiation to advocate for potentially therapy-altering evaluation of tumor samples. Our hope is that providers of all Canadian children with brain tumors now have access to written guidance to provide equivalent care across the country for the majority of CNS tumors.

## Data Availability Statement

The raw data supporting the conclusions of this article will be made available by the authors, without undue reservation.

## Author Contributions

JuB, BC, and UB organized the writing of each section. JuB, SC, HC, CE, AFo, JH, DJ, LLC, AL, VR, IV, and SZ contributed to writing of each section. JuB and UB prepared the manuscript. All authors contributed to the article and approved the submitted version.

## Conflict of Interest

The authors declare that the research was conducted in the absence of any commercial or financial relationships that could be construed as a potential conflict of interest.

The reviewer SL declared a past co-authorship with the authors to the handling Editor.
